# Use of low-power He-Ne laser therapy to accelerate regeneration processes of injured sciatic nerve in rabbit

**DOI:** 10.1186/s41983-018-0047-6

**Published:** 2019-01-05

**Authors:** Ahmed Majeed Al-Shammari, Yahya Syhood, Ahmed S. Al-Khafaji

**Affiliations:** 1 Experimental Therapy Department, Iraqi Center for Cancer and Medical Genetic Research, Mustansiriyah University, Baghdad, 1001 Iraq; 2 Department of Biology, College of Science, University of Baghdad, Baghdad, Iraq

**Keywords:** Peripheral nerve, Nerve healing, Photostimulation, Wallerian degeneration, Nerve injuries

## Abstract

**Background:**

Photostimulation using low-power laser had been used for nervous repair with interesting results. This study aimed to evaluate the influence of 20 mW low-power He-Ne laser on the regeneration of a peripheral sciatic nerve after trauma using the Albino rabbit as an animal model for experimental treatment.

**Methods:**

Six adult male rabbits were randomly assigned into two equal groups (control- and laser-treated). General anesthesia was administered intramuscularly, and exploration of the sciatic nerve was done in the lateral aspect of the legs. Complete longitudinal and reverse sections of the nerve were performed, which was followed by crushing of the neural sheath. Treatment was carried out directly after the trauma. Irradiation doses of low-level laser therapy (LLLT-31.5 J/cm^2^**)** with once a day application for 10 consecutive days and observed for 30 days. The animals were followed up for an extra 2 weeks. Two important factors were examined histopathology and functionality of the nerve.

**Results:**

Compared to the control group, significant variations in regeneration were observed, including thicker nerve fibers, and more regular myelin layers in the treated group.

**Conclusions:**

The results of the present study suggest that laser therapy may be a viable approach for nerve regeneration and repair.

## Introduction

Peripheral nerves traumatic injuries that may result in substantial disability are a universal problem. In periods of peace, it is caused by motorized vehicle accidents, and less frequently from sharp objects, falls, and work-related accidents [20]. In countries that suffered from years of wars and conflicts such as Iraq [3] could have more peripheral nerve injuries resulted from explosions, bullet injuries, and many other reasons that need attention and treatment [19]. Nerve injuries that include complete transection of the nerve results in degeneration occurs both distal to the lesion segment by Wallerian degeneration and proximal to the lesion segment by retrograde degeneration [17], which lead to motor and sensory functional damage at the spot of the lesion. While some regeneration can be seen in nerves where lesions were untreated, the progression is usually slow and mostly inadequate [13]. This regenerative incapability proves the necessity for suitable treatment to accelerate and improve the nerve repair progression.

Investigation of low-level laser therapy (LLLT) in the clinical applications has been done in different fields, and it was used to relieve pain and promote the recovery of several pathological conditions [8, 14]. LLLT has photobiomodulating effect, which can stimulate tissue metabolism. The mechanism involves absorption of the photoreceptors to the photons, leading to changes in the ATP synthesis in mitochondria by accelerating the transport chains of the electrons, thus modifying cell responses [18].

Peripheral nerve injury (PNI) animal models have been established to estimate the outcome of LLLT in the regeneration of injured nerves [4]. Previous studies, which evaluated the effects of low-power laser irradiation on rats crushed injured peripheral nerves, revealed protecting instant effects which enhance the functional activity of the injured peripheral nerve [23]. Moreover, it help to maintain the functional activity of the injured nerve over time [21], increase myelin sheath thickness [24], as well as the axonal diameter [16], induce Schwann cells proliferation [7], and induce neurotrophic growth factors expression [12]. Furthermore, laser is used with photosensitizers for cancer treatment [1, 2].

The aim of this study is to explore the effectiveness of He-Ne laser as LLLT to help neural regeneration in a rabbit sciatic nerve injury model.

## Materials and methods

### Ethical statement

The experimental procedures followed the guidelines for the ethical treatment of experimental animals and were approved by Mustansiriyah University, Iraqi Center for Cancer and Medical Genetic Research, Animal Care, and Use Ethics Committee. The date of ethical approval is 30 July 2010, and reference number was 5.

### Experimental animals

The animals used were 8 New Zealand White rabbits (*Oryctolagus cuniculus*), adult male (weight 2.8–3.9 kg) and 3 months old. The animals were randomly divided into two experimental groups, four in each: first group treated with LLLT at dose of irradiation (31.5) J/cm2. The second group was control non-irradiated. Both groups received a standard diet and water during the study and were kept under standard lighting, temperature, and humidity environment.

### Anesthesia

All rabbits were anesthetized by xylazine hydrochloride 2% (5 mg/kg body weight) and ketamine hydrochloride 10% (50 mg/kg body weight) through intramuscular injection.

### Surgical procedure

The experimental surgical procedure was performed according to La et al. [15] under the typical aseptic conditions via exposing the sciatic nerve through midline incision at the back of the right leg and longitudinal separation of the muscles. Focal crushing of right and left sciatic nerve of each rabbit was done for 60 s by straight hemostat. The wound was closed using silk simple sutures. The surgical procedures were performed by a veterinary surgeon.

Postoperatively, 40.0 mg gentamycin were given to the rabbits for 4 days to prevent infection. Clinical assessment was measured postoperatively via recording the daily examination of ability onset to walk after operation until 2 months.

### Laser irradiations

**Fig. 1:**
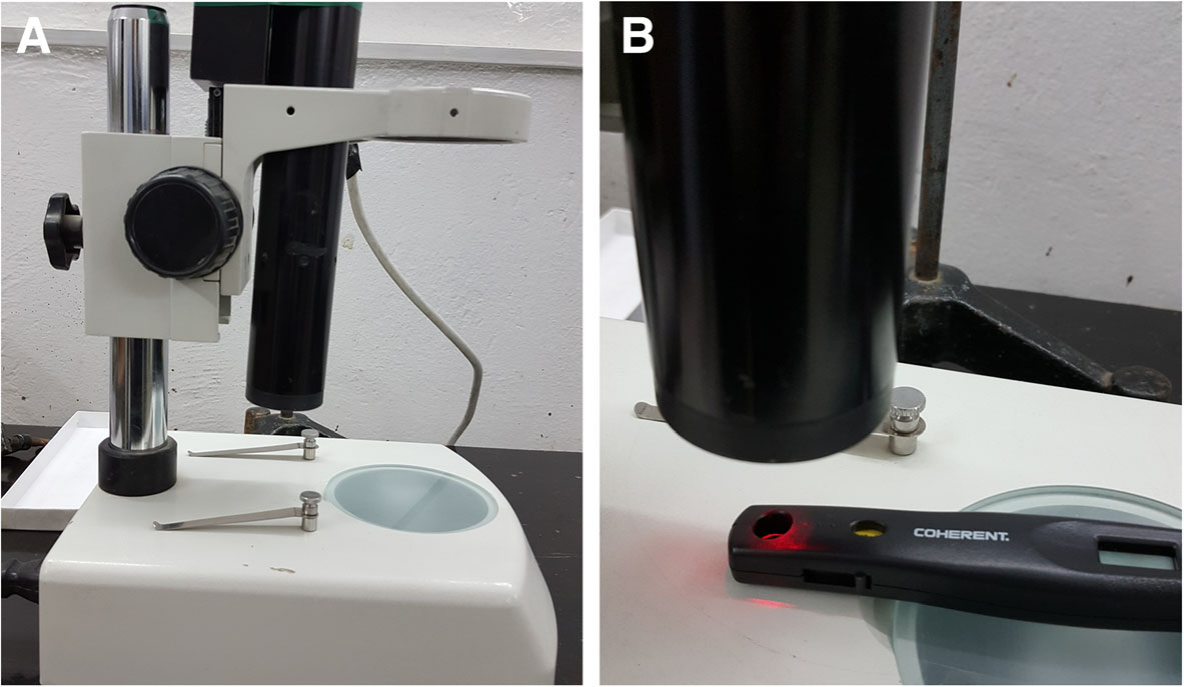
**a** The source of laser used in this study was Helium-Neon (He-Ne) gas laser (Model DL30, LG Lasers) with output power approximately 20 mW as a continuous wave irradiation. **b** The wavelength of light that emitted from this laser and output power were determined by using a portable power meter (LaserCheck, Coherent CA, USA)

The source of laser used in this study was Helium-Neon (He-Ne) gas laser (Model DL30, LG Lasers) with output power approximately 20 mW as a continuous wave irradiation (Fig. 1a). The wavelength of light that emitted from this laser and output power were determined by using a portable power meter (LaserCheck, Coherent CA, USA) (Fig. 1b), according to the measurements, the wavelength was 630 nm, penetration of 0.6 cm. The irradiation area was (0.125) cm^2^, while the exposure time of irradiation was 240 s, Dose of irradiation was 31.5 J/cm^2^. The treatment by laser was started first day post-surgical, where all the rabbits in the treatment group were exposed to the identical technique with once a day application for 10 consecutive days and observed for 30 days.

Rabbits were handled softly, and laser treatment did not yield any painful impression to the treated group. Control group were exposed to the same technique, but with no laser treatment.

### Gross examination

Clinical assessment was measured postoperatively via recording the daily examination of ability onset to walk after operation until 30 days.

### Euthanasia

All animals were sacrificed 30 days post-surgery using overdose of anesthesia, and the healed wound nerve segment of the operated location was collected from each sciatic nerve.

### Histopathological examination

Every 7 days of treatment, one longitudinal section was made through the site of repair and was stained with hematoxylin and eosin. They were then examined under light microscopy for qualitative assessment of the repair process. Transverse sections were obtained for quantitative assessment of the diameters of the axons.

Directly after harvesting the repaired nerves, they were fixed in 10% neutral buffer formalin, and the specimens were sent for histological processing. The samples were treated with xylene, dehydrated using graded ethanol, nerve tissue samples were paraffin embedded, sectioned at 5 μm longitudinally, via microtome and stained with hematoxylin-eosin. The injured nerve area was observed under a light microscope (Leica-microsystems, Germany).

### Histopathological images quantitative analysis

The histopathological sections were photographed at _× 200 magnification at four randomly designated fields at the histological sections, using the light microscope (Leica-microsystems, Germany) and a digital color camera (Motic, Hong Kong). The pictures were analyzed using ImageJ software (http://rsb.info.nih.gov/ij/). The longitudinal and transverse sections of the nerve samples were analyzed by pathologist. The histopathology examiner was unaware about the groups. For statistical analysis, the quantitative measurement of each picture was taken at least three times. Nerve nuclei and vacuoles (%) in sections stained by hematoxylin-eosin (H&E) were measured. This was done according to Yang et al. [30].

## Results

**Fig. 2:**
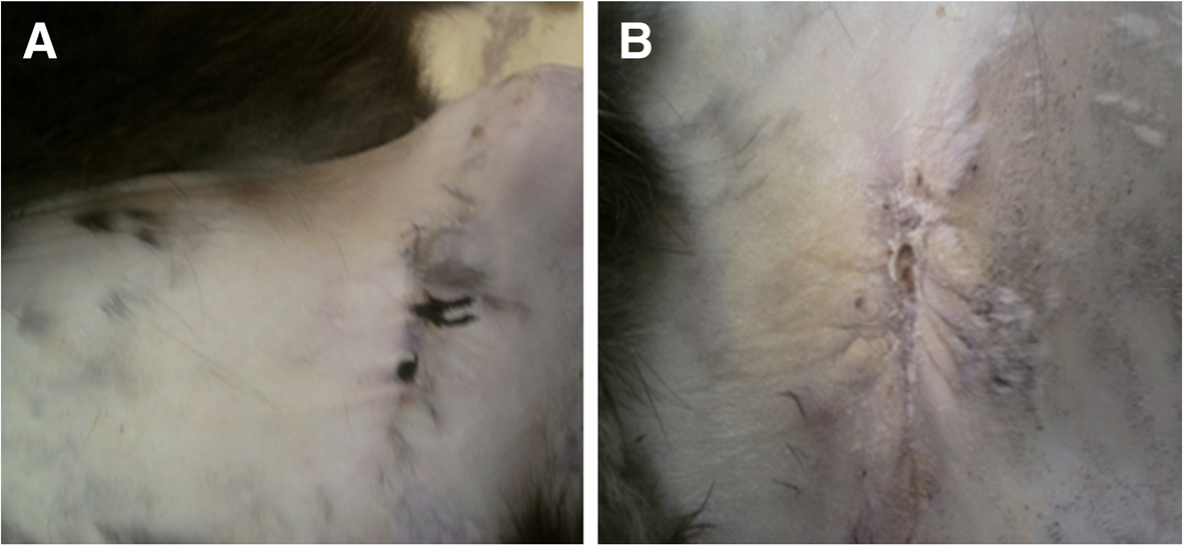
**a** Crush site of sciatic nerve in the treated group. **b** Untreated rabbit wound site

The current experiment evaluated the regenerative capacity of nerve repair by red LLLT irradiation for 10 days. All the animals well tolerated the surgery and lived until the end of the experiment with no postoperative complications, infections, or wound openings detected. The gross examination showed that, in both groups (control and treatment), the injury site could still be identified. In the laser treated group, there was enhancement of functionality after 30 days of treatment where the site of crushing was healed grossly (Fig. 2a, b). The ability of walk is better than untreated control group.

**Fig. 3:**
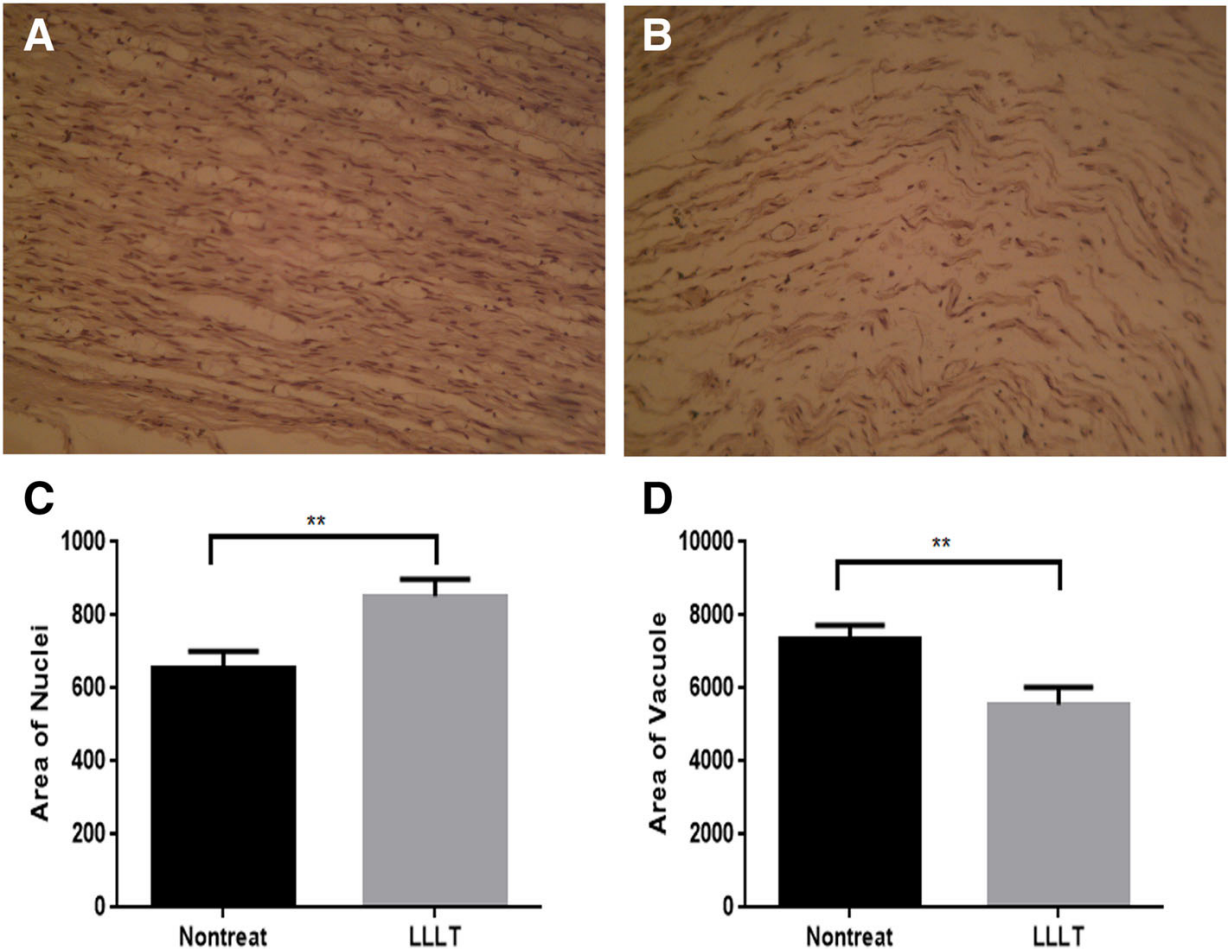
Histopathological examination. **a** Longitudinal sections through the site of repair exhibited sprouting of the axons and enhanced alignment of nerve fibers at the site of laser irradiation. 100× H&E. **b** Untreated control nerves revealed loss of axons (100×) H&E. **c**, **d** Longitudinal sections of sciatic nerves quantitative assessments for nuclei and vacuole formation. Sections of sciatic nerves gained from injured rabbits with non-treated and LLLT treatments. Quantitative analyses for Schwann cells nuclei and vacuoles formation showing enhancement in repair and healing for the treated nerve with significant changes between each other at a confidence level of *p* < 0.05. (H&E)

**Fig. 4:**
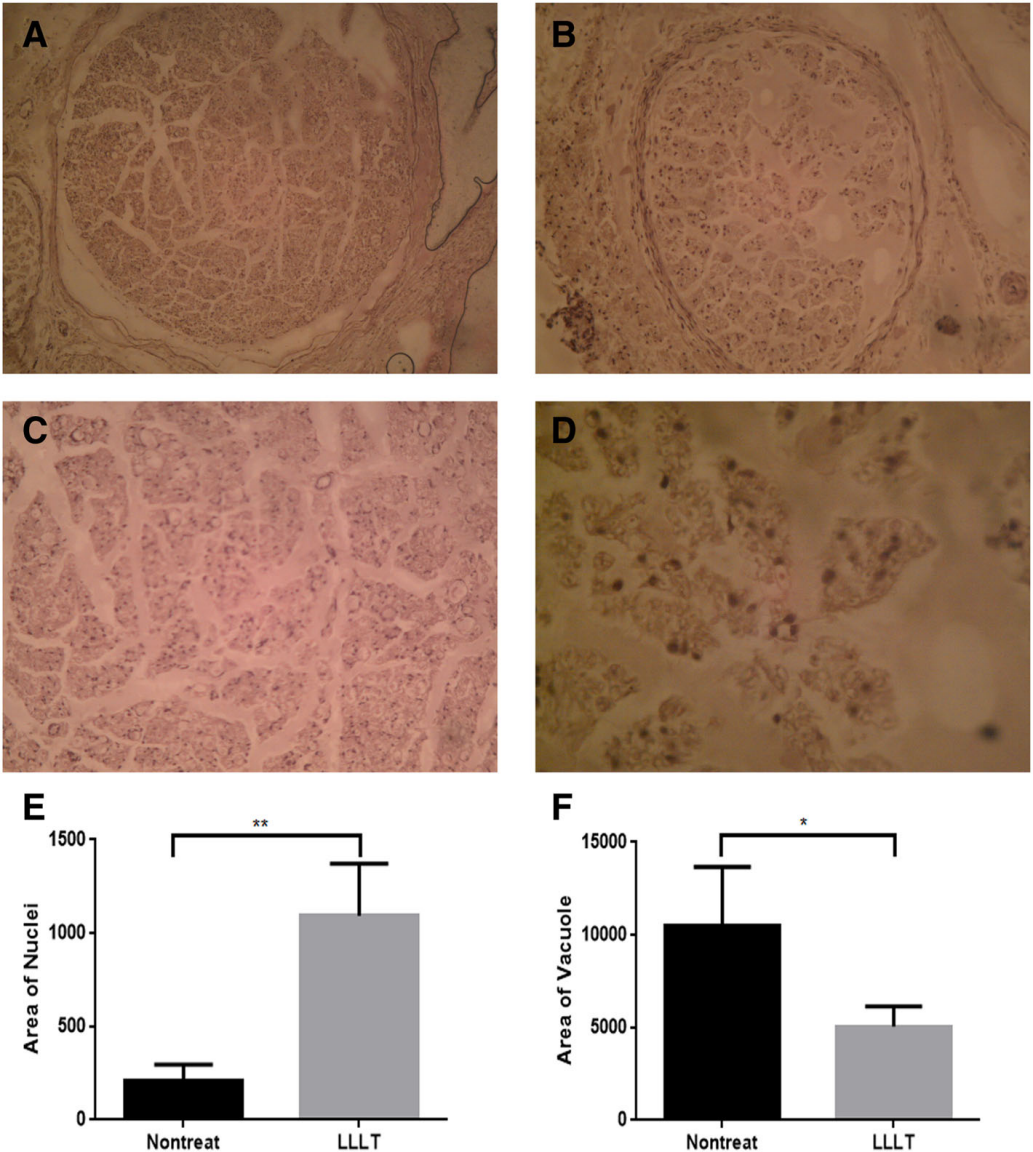
Histological images of transected sciatic nerve after hematoxylin and eosin staining in the treatment (LLLT) group (**a** and **c**) and untreated group (**b** and **d**). **a**, **b** Lower magnified views of the LLLT and no treatment groups. The transverse sections showed Wallerian degeneration in the untreated group. While nerve fibers and Schwann cells numbers increased in the treated group. **e** and **f** Histopathological pictures quantitative analyses for nuclei and vacuole formation showing remarkable higher number of Schwann cells nuclei and lower number of vacuoles referring to healing for the treated nerve with significant changes between control and treated groups at a confidence level of *p* < 0.05

Histopathological examination of longitudinal sections through the site of repair showed prevalent sprouting of the axons and enhanced parallel alignment of nerve fibers at the laser irradiation site. While inspection of the tissue section of the untreated control nerves revealed loss of axons, with more noticeable formation of fibrosis (Fig. 3b). Histological transverse section of sciatic nerve in treated group (Fig. 4a, c) revealed improved aspect of segments with large and small nerve fibers with myelin sheaths of proper thickness. Histological investigation showed uninterrupted epineurium in the laser-treated nerves. There was no inflammatory reaction to the laser treatment was seen in nerve sections. Untreated control group (Fig. 4b, d) Showing swollen and degenerated fibers with thin myelin sheaths, experiencing Wallerian degeneration (Fig. 4d). The laser-treated nerve displayed an increased quantity of nerve fibers, Schwann cells, and myelinated axons compared to the untreated nerve.

### Morphological changes improvement by LLLT therapy

The morphological changes parameters, for instance vacuoles and the nuclei numbers, were presented in Figs. 3 and 4. Nuclei numbers in LLLT-treated nerve were higher than untreated nerve, showing more Schwann cells after nerve crush treated with laser when compared with non-treated nerve (Figs. 3a, b and 4a, b). The formation of vacuoles was increased as well after crushing the sciatic nerve. Laser administration reduced the crushing-induced vacuole formation event in comparison to non-treated nerve (Fig. 4c, d). Statistical analysis to the increase cellularity of treated nerve as represented by increased number of Schwann cells nuclei were significant (*p* < 0.01; Figs. 3c and 4e). The LLLT treatment significantly reduced vacuole formation event, *p* < 0.01, Figs. 3d and 4f).

## Discussion

In the present study, we showed that 630-nm-Helium-Neon (He-Ne) gas laser at a dose of 31.5 J/ cm^2^ induce substantial functional and histological recovery in rabbit-crushed sciatic nerves. Laser therapy is a practical phototherapeutic method with investigational evidences for the peripheral nerve injury treatment, indicating encouraging effects on the neuromuscular repair process that induce functional indices improvement, and increase cytokines and growth factors expression [5, 10]. It also found to enhance the speed of treated nerve conduction [27]. The animals were sacrificed after completion of all experimental studies on the last day of the 30 days period for analysis of nerve histology as described by several studies as sufficient time for the experiment [28, 30]. One of the study limitations is lack of multivariate regression analysis due to small sample size.

Histological findings observed in current experiment with laser therapy were exceptionally improved than those noticed in control untreated nerve, as the quantity of fibers decreased, while it increased progressively in laser irradiated nerves. Schwann cells also increased in number due to stimulation via laser treatment. Consequently, the current study confirmed the efficacy of He-Ne laser for the peripheral nerve injuries treatment, by means of the histopathological assessment for showing nerve regeneration after treatment. Takhtfooladi and Sharifi [28] observed a significant histological alteration after low-laser therapy for the increase of neurons and Schwann cells number leading to efficient treatment. Barez et al. [6] showed that treated nerves exhibited reduction of Wallerian degeneration, increase Schwann cells number, and regeneration of the injured nerve starting from the third day of surgery. He-Ne laser irradiation described by Van Breugel and Bär [29] induce a positive effect on sciatic nerve regeneration via direct effect on Schwann cells by experimental investigation. They cultured Schwann cells from sciatic nerves in vitro and irradiated them by a He-Ne laser beam of 632.8 nm leading to significant proliferation of Schwann cells, compared to non-irradiated control cells. Several studies showed that low-energy He-Ne laser significantly restores crushed sciatic nerves in rats and other animal models [21, 22, 26].

Quantitative and morphometric results showed significant improvement after laser irradiation in comparison to the untreated control nerve. Ziago et al. [31] treated crushed sciatic nerve of rats via low-level laser therapy and showed enhanced morphometric data in the LLLT group compared to the control untreated group. Histomorphometric assessments by Shen et al. [25] prove that the LLLT treatment augmented peripheral nerve injury repair. Similar to current experiment results about reduction in vacuoles number [30] demonstrated less vacuole formation after crushed nerve LLLT treatment.

Laser therapy in vivo studies accelerate and enhance the regeneration of the injured nerve tissues and effective in restoring sensitivity. In order to standardize procedures for clinical use, clinical trials should be conducted [9]. Recent review followed several studies on different laser types (He-Ne, Diode, GaAlAs) with 660–860 nm wavelength range and 20–250 mW radiation power, 0.45–70 J/cm2 energy density to treat patients via LLLT found to enhance patients’ sensory function [11].

## Conclusion

Low-power He-Ne laser irradiation (632 nm) positively influenced the regeneration of severe crushed injured sciatic nerve in rabbits. Therefore, it can be valuable for treating many pathological lesions of peripheral nerves in clinics.

## Abbreviations

H&E: Hematoxylin and eosin
He-Ne: Helium-Neon
LLLT: Low-level laser therapy
PNI: Peripheral nerve injury
